# Supplementary material for: Qualitative evaluation of a pilot mental health program for public safety personnel with post-traumatic stress disorder
Source: PLOS Ment Health. 2026 Jan 30;3(1):e0000536. doi: 10.1371/journal.pmen.0000536 (PMC12857991; doi:10.1371/journal.pmen.0000536)
Supplement: S1 File — (DOCX) [file pmen.0000536.s001.docx]

# S1 File: Interview guides

## Interview guide clinical staff

Background

1. Can you tell me a bit about your clinical background and training?
2. How would you describe your work experience with the first responder population?
3. Can you tell be about your role in the FRMHT program?
4. Can you describe the FRMHT program as you understand it? *Prompts:* *What makes it different from other MH treatment programs? Why was it implemented? How is it tailored to first responders?*

Program Evaluation

1. Who do you think would benefit most from the FRMHT program? *Prompts: In terms of (professional) background? Type of trauma? Length of time off from work? Personal circumstances (support from family, friends)?*
2. What are you looking at when you consider whether a client is appropriate for this program? *Prompts: Are there specific trauma histories? Treatment histories? Personal characteristics?*
3. In your view, how do the therapeutic needs and challenges of first responders differ from a more general population who suffer from PTSD? *Prompts: Do you see a difference in the nature of the trauma? Does it affect them in a different way (professionally/personally)?*
4. What do you see as the best way to treat FRs suffering from PTSD?
5. What is your goal when treating FRs as part of the FRMHT program? *Prompts: What do clients generally have as their main goal?*
6. From your perspective, why do you think FRs might like the FRMHT program? What do you think they struggle with?
7. Can you describe a case where you felt the FRMHT program worked well? *Prompts: What about this case do you think was crucial for success?*
8. How do you think the FRMHT program should be offered to first responders? *Prompts: When would you recommend a first responder try the program? Should it be available immediately when they are diagnosed with PTSD? Even earlier? Are there treatments FRs should try first?*
9. In your experience, what might make a client leave or not finish the program?
10. What do you see as the main struggle for FRs with PTSD in preparing to return to work? *Prompts: Is the nature of the work concerning? Is the culture of the workplace concerning? What kind of follow-up support do they need? What alternatives would you suggest? And when?*
11. Broadly speaking, how do you think the FRMHT program could be improved? *Prompts: Are there aspects about its structure? Mode(s) of delivery? Referral and assessment process? Its goals? Does it reach all people who could benefit?*

Concluding

1. What aspects of the program that we have not talked about do you think are important to consider when thinking about how it could be improved?

## Interview guide Public Safety Personnel

Thank you so much for being willing to share some of your experiences with us. [*Explain that the interview is confidential and remind the participant that their confidentiality and their right to withdraw consent will be respected at all times. Remind the participant that they can ask for a break at any time if they would like one, and that we can end the interview at any time if they experience distress, discomfort or for any other reason. Questions will not be about their trauma directly, but their experience with different treatment programs. Explain that you will provide warnings for questions that may be distressing and that they can skip these questions. Explain that the interview will help the research team better understand how the mental health treatment program can help first responders who suffer from PTSD.*]

**The first questions will be about your time as a first responder.**

1. Can you tell me a bit about your job? *Prompts: What type of first responder are you (e.g. firefighter, police, ems, other)? How long have you been a first responder? What do you like about the job?*
2. What do you see as the main challenges of your job? *Prompts: How are the working hours? The physical demands? Do you see any negative impacts on your personal life? Have you had to take time off previously either because of your physical or mental health?*
3. How would you describe the culture at your job around the mental health impact of the work? *Prompts: Does the management have some kind of policy? Do you feel you are able to speak freely about your experiences with others such as colleagues or supervisors? Why (not)?*

**Next I will ask some questions about the treatment you have received. Just to remind you that if at any point you feel uncomfortable, we can take a break or stop the interview. If you’d rather not answer a question, we can skip to the next one.**

1. Were you aware of the potential mental stress and trauma of the work? *Prompts: Were you provided information before you started your job? Did this play a role in your career choice?*
2. What, if any support programs were available to you as a first responder? *Prompts: Did your employer provide support programs (e.g. counselling, support groups)? How do you feel about the supports that are provided? Did you seek out opportunities?*
3. Was there follow-up after potentially traumatic events? *Prompts: Were you or colleagues referred for counselling or support programs? Were you offered other services?*
4. Could you talk me through how you came to have to take time off work? If there are things you’d rather not talk about, you can leave those out. *Prompts: Was there a specific moment you noticed that you struggled? Did you reach out for support from supervisors, colleagues, friends/family, or elsewhere? Where there intermediate steps? Was it hard to secure a compensation claim? Did parts of the process cause additional stress?*
5. How would you describe the support you received from your employer once you took sick leave? *Prompts: Did they help you find treatment programs? Did you feel they understood your situation? Did you feel pressured in any way to return to work?*
6. Could you describe your experiences with any treatment programs before you were referred to the FRMHT program? *Prompts: How were these programs set up? Why did you feel these programs were not helpful?*
7. Why did you decide to participate in the FRMHT program? *Prompts: Who suggested the program to you? What convinced you to try it? Did you have any expectations beforehand?*
8. Could you describe your experience with the FRMHT program (so far)? *Prompts: How was the referral and assessment process? What did you think about the sessions? Did you consider stopping at any point? If so, why? Are there aspects you would have liked to do differently?*
9. What do/did you hope to achieve with the FRMHT program? *Prompts: Return to your work as a first responder? Transition to a different career? Improvements to everyday life? Techniques to help you manage or overcome your trauma?*
10. Did you achieve the goals you hoped / How do you feel you are progressing towards your goals? Why? *Prompts: What aspects of the program did you find helpful? Which ones were not helpful to you? Did the program help in other, unforeseen ways?*
11. If you have previously participated in other PTSD programs, could you provide examples of how the FRMHT program is different from other PTSD treatment programs you have seen or participated in? *Prompts: What makes it more suitable for people in your line of work?*
12. Even if the FRMHT program helps you reach the desired outcomes, what do you see as the main challenges for returning to work – either your previous job or a new job? *Prompts: Do you worry about the opinions of colleagues or supervisors? The pressure of the job? Your ability to cope with potentially traumatic situations? When would you feel ready to return to work? Irregular working hours?*
13. After finishing the FRMHT program, what kind of additional support would you like to have? *Prompt: What would help you return to work? What would you need in terms of on the job support?*
14. How do you view the overall process of taking time of work, getting help and up to the point of potentially returning to work? *Prompts: Do the various people and organisations involved have a fair approach (e.g. employer, clinical staff, caseworker)? Are there situations where you felt that you weren’t understood? Do you feel you were provided adequate time and opportunities to work through your experience?*
15. Would you recommend the FRMHT program to colleagues who have experienced trauma? *Prompt: If not directly, do you think your employer should refer your colleagues to the program?*

Concluding

1. Is there anything about the FRMHT program that we have not discussed that you feel are important for us to consider, when we are trying to make it better for other first responders suffering from PTSD?

Thank you so much for your participation. We’re very grateful to you for sharing your experiences.

**Include appropriate support resources, as well as contact info for appropriate hospital personnel if respondent experiences emotional difficulty during the interview, or wishes to provide further feedback to the hospital.

These interviews are anticipated to be approximately 60 minutes in length and will explore the experiences of the First Responders who have participated in the programs, whether and how they feel they benefited from the programs, what elements are perceived to be unique/different for First Responders, what participants think should be changed about the programs and what prospects they think they have for a return to work and what kinds of accommodations they think they would need in order to be able to return to work successfully. Consideration for the type of First Responder and perceptions of workplace culture will also be considered in the interviews.
