# Supplementary material for: Qualitative evaluation of a pilot mental health program for public safety personnel with post-traumatic stress disorder
Source: PLOS Ment Health. 2026 Jan 30;3(1):e0000536. doi: 10.1371/journal.pmen.0000536 (PMC12857991; doi:10.1371/journal.pmen.0000536)
Supplement: Attachment — Submitted filename: PLOS Mental Health - Response Letter DEC 06.docx [file pmen.0000536.s003.docx]

| PLOS Mental Health  Dr Lambert Zixin Li | Dr Lucas Seuren  [Lucas.seuren@thp.ca](mailto:Lucas.seuren@thp.ca)  Institute for Better Health  Trillium Health Partners  100 Queensway West  Mississauga, ON L5B 1B8  Canada |
| --- | --- |

December 5, 2025

**Re: PMEN-D-25-00363**

**“Qualitative evaluation of a pilot mental health program for public safety personnel with post-traumatic stress disorder”**

Dear Dr Li,

We want to thank the reviewers for their extensive and detailed feedback on our manuscript; this has definitely helped us with making revisions and improvements. In accordance with your request, we have enclosed a point-by-point discussion of the changes we made in relation to the comments of the reviewers as well as both a manuscript with and one without track changes.

We have attempted to include as much of the reviewer comments as possible. At times, this involved balancing divergent viewpoints from different reviewers. The extent of the suggestions also meant that we could not incorporate them all, as that would lead to an excessively long manuscript. For those cases, we chose to focus on those comments that were most in line with the aim of the paper and the audience we had considered – for example, we focused less on psychology and traumatology than on implementation science, even if we did try to incorporate those viewpoints where possible.

Best wishes,

Lucas Seuren

Reviewer #1: 

| I recommend briefly explaining the distinction between the terms PTSD and PTSI and why PTSI is used in the context of PSP. | We had originally chosen to use the wider term PTSI throughout as it aligns with efforts at reducing stigma through emphasizing work-related injury that can be treated, but as the program is specifically designed and advertised to PSP who are on leave with PTSD, we have changed the specificity to PTSD where appropriate. We retain the use of PTSI more broadly were appropriate which helps to capture the complexity of treating patients with comorbidities, as we see in the results. For example, some participants spoke of mental health challenges other than PTSD in their groups so we sometimes reference those discussions by using the broader term PTSI. |
| --- | --- |
| Line 74: How can the large range be explained? | We added a line explaining potential variation due to methods, region, etc. |
| The symptoms of PTSI should be presented and explained why they can potentially render a person unable to work. | This is a good point and we have added some symptoms to the Introduction of the paper. |
| Why was no established qualitative method used as a basis, e.g., Qualitative Content Analysis (according to Mayring or Kuckartz)? | We understand the reviewer’s question. We felt that Descriptive Analysis as defined by Colafari & Evans (2016) and Neergaard et al (2009) was a better approach given the focus on developing recommendations for improvement. This allowed us to make interpretations, but stay close to the practical concerns of PSP and mental health staff. |
| How was the interview guide created? Deductively from the framework? Were inductive codes also generated from the text material? | We have updated our Data Collection section to provide more detail on how the guides were designed. We used three concepts from Proctor et al to develop questions shaped for each specific group, so deductively. As we explain at the start of the Analysis section, data were analyzed inductively with open coding, while using the three core categories as sensitizing concepts. |
| To what extent does the interview guide differ from the framework developed by Proctor et al.? | We used their definitions to help us draft relevant interview questions. Relevant experience and expertise from the broader team then informed revision of the guides. There is no standardized list of questions within their framework. |
| Who created the interview guide? Was this reflected in a collegial exchange? | We now state who created the interview guides and how this was done in Data Collection section. |
| Line 192: What exactly are the interviewers' areas of expertise? | We have added a line to note L.S.’s expertise in Implementation Science. For A.B. the ms mentions her expertise in occupational and posttraumatic stress and interviewing trauma-exposed PSP. |
| Were notes taken during the interviews? | We have added this to the manuscript |
| I recommend making the code system available in the appendix. | We understand the reviewer’s request, but we did not have a standardized coding system, as we used an open coding approach. So open codes were categorized into overarching themes. |
| What rules were used for recruitment? (Selective sampling? Maximum variance?) | We now state more explicitly how participants were recruited and discuss the associated limitations in the Discussion section. |
| What criteria were used to end recruitment? | We added that recruitment ended because the study ended. |
| To increase transparency, I recommend attaching the COREQ checklist as a supplement: Tong A, Sainsbury P, Craig J. Consolidated criteria for reporting qualitative research (COREQ): a 32-item checklist for interviews and focus groups. International Journal for Quality in Health Care. 2007. Volume 19, Number 6: pp. 349 – 357 | We appreciate the reviewer’s point that this would provide more transparency, and we have included it for the reviewers. However, we do not think it offers value for readers, as the checklist merely shows where in the paper these aspects are reported: the material is in the manuscript. |
| When was the coding process completed? Was content saturation achieved? If not, please explain why and include this in the limitations section if applicable. | These are helpful comments. We added how we conducted and completed the coding process, and what saturation meant in this paper. We have also added this to the limitations of the study (no participant saturation, only within sample saturation). |
| To what extent were the quality criteria taken into account? Was countercoding used? | We have expanded in the Analysis section on how we aimed to preserve academic rigour such as negative case analysis. Counter coding was inherent to the sensitizing concepts. For example, virtual delivery was coded as both providing safety and lacking connectivity. We also expanded in the Limitations section on how this could be improved. |
| Lines 186/187 are unclear: who are the “staff involved in the comprehensive assessment”? | We see this was unclear, and we have rewritten it to show that it was staff conducting the comprehensive assessments prior to treatment commencement. |
| Line 199: Shouldn't it say ‘effectiveness’ instead of ‘acceptability’? | Thank you for correcting this. |
| The analysis process should be described in more detail: How was the text processed to increase the level of abstraction? Were memos created? Were inductive codes created? | We have expanded on the Analysis section. As mentioned above, the Analysis section states at the start that the coding was done inductively using open coding as well as the sensitizing concepts of appropriateness, acceptability, and effectiveness. |
| Line 212: Does a nurse belong to the PSP group? | Yes, in Ontario, nurses who provide direct patient care are included in the list of professions where a diagnosis of PTSD is presumed to be work-related, making them eligible for the treatment program. |
| I recommend reporting which aspects were mentioned very frequently in the interviews | We agree that showing which topics were mentioned most frequently could provide evidence in some more deductive coding approaches such as deductive content analysis. However, as we used an inductive and interpretive approach to support exploration, frequencies were not recorded. Thematic findings however, do largely report on aspects of treatment that were discussed across both PSP and staff, inferring some level of commonality rather than single occurrences. |
| Overall, the discussion still lacks depth! | We agree with the reviewer that our previous Discussion was mostly just restating the Results section. Using the comments from all reviewers, we have revised the section. |
| At the beginning: a brief summary of the results. | Our Discussion now starts with a summary of the key findings. |
| I miss key terms from psych traumatology that can be discussed very well based on the quotations, e.g.: -second-victim phenomenon -resilience -bloc-building effect  -avoidance behavior -safe space | We see the value of these terms for treatment programs, and we have attempted to integrate them where appropriate. However, as none of the authors are mental health clinicians, our focus was more on practical implications , and we do not believe we are well positioned to address these topics in depth. |
| On the question of effectiveness: The timing of the survey is significant from a traumatological point of view because processing traumatic content takes time (several months!). This means that if the survey is conducted too soon after the intervention, it may not be possible to evaluate its effectiveness. In addition, the participants expressed that psychoeducation is an important aspect in dealing with the symptoms. | This is a very interesting point that would clearly be valuable to how effectiveness would be assessed. Interviews were conducted at variable lengths after discharge (weeks to months). As we lack follow-up data, we do not know long-term effects, but we did include a reference to a quantitative analysis of the program which shows no effect in terms of successful return to work, which could be related to this issue. As we do not provide survey data in this paper we have not expanded further. (The quantitative paper had not yet been submitted at the point when we submitted our manuscript.) |
| It would be interesting to know whether the questionnaires were completed by the participants themselves or by the therapists (bias of socially desirable responses!?) | We assume the reviewer meant the clinical questionnaires that PSP filled in during the treatment. This is an interesting point, and we have added this to the Methods section. |
| Line 580: This is a recruitment bias and should be identified as such, as only those who are fundamentally open to therapy participate. | We agree with the reviewer and now explicitly state that it’s a bias. |
| Comorbidity: This low-threshold offer may not be suitable for taking comorbidities into account. However, it is still justified because it prevents post-traumatic stress from becoming chronic. My recommendation would be to emphasize prevention rather than therapy in the discussion. | We see the reviewer’s points. We have condensed our discussion of comorbidities in line with the anecdotal evidence. We also included a mention of prevention, albeit in the larger context of supporting PSP. |
| Formatting: Some of the quotations are not indented (e.g., line 392). | We appreciate the reviewer’s careful look and have corrected this |
| The PSP quotes suggest that training peers who are familiar with the work environment and terminology can be useful in increasing acceptance. | We agree that this could be a useful improvement. We now have a separate section on Recommendations, where we discuss this more explicitly. |
| The social aspect could possibly be discussed: the public should be interested in the mental health of their security personnel. That is why such tailor-made programs are very valuable! | This is a really interesting point. While we lack adequate space for complete discussion, we did add this to the Conclusion. |
| The weaknesses of the program are clearly evident from the quotations. A concluding presentation of the aspects that should be improved would be desirable. | As mentioned above, we have now made more explicit recommendations. |

Reviewer #2:

| Strengths:  1. The study fills a gap by comparing PSP and provider perspectives, which is rarely done.  2. The paper offers policy and programmatic implications for implementation of trauma-informed care.  3. The three organizing constructs (appropriateness, acceptability, effectiveness) are well-applied. | We want to thank the reviewer for their kind words. |
| --- | --- |
| 1. The introduction describes PSP vulnerability well, but it reads more as an extended literature review than a conceptual framing. Consider adding a short paragraph near the end of the Introduction linking PSP treatment experiences to implementation science and cultural competence theories (e.g., Normalization Process Theory, or cultural safety frameworks). | We see the reviewer’s point that our Introduction only focused on background and not on conceptual framing of the paper. We have added a short paragraph explaining implementation science, how it matters, and we explain what a formative evaluation is. |
| 2. Currently, “credibility” emerges inductively in findings but is not theoretically introduced—yet it becomes the linchpin concept. Introducing “credibility” as a theoretical construct (trust, legitimacy, cultural safety) earlier would improve coherence. | This is a valuable point, given how central credibility is to our analysis as an inductive finding. We have fronted this point to the start of the Results section and made it more central in the Discussion |
| 3. The methods mention that one interviewer had trauma-related expertise but not PSP lived experience. Reflexivity could be explicitly discussed e.g., how researcher background, gender, or institutional affiliation may have influenced interviews or interpretation. | We added some further reflection to the Methods, Discussion and Limitations around how the lack of direct PSP lived experience for the interviewers, as well as potential sociodemographic factors, may have shaped the interviews. |
| 4. Specify how saturation or theme stability was determined. | We now explicitly state this in the Methods section and discuss limitations around Saturation in the Discussion. |
| 5. The analysis is rich but largely descriptive. Some findings could be elevated with modest interpretive commentary (e.g., the “abrupt discharge” theme could be tied to continuity of care literature or models of recovery capital; and “virtual delivery” could engage more critically with the literature on digital relationality and trauma therapy). | We appreciate the suggestions from the reviewer. In line with comments from some of the other reviewers, we have aimed to provide a more interpretive Discussion section, tying our descriptive analysis to broader literature (including continuity of care). |
| 6. Clarify distinction between prevalence of PTSI and exposure to PPTEs to avoid conflating them. | We have attempted to make this distinction explicit at the start of the Introduction. |
| 7. The framing of “effectiveness” is somewhat ambiguous. It oscillates between program evaluation sense and participant perception. Clarify early that the study examines perceived effectiveness. Similarly, “formative evaluation” should be briefly defined and distinguished from summative evaluation. | We see the problem with these interpretations – especially as the focus is often on quantitative metrics of effectiveness. Our definition of the concept explicitly uses the term perceived, and we have attempted to keep this clearer. We have also followed the reviewer’s recommendation and explained how formative and summative evaluations differ. |
| 8. There are several repeated citations (e.g., Haugen et al., 2012; Lewis-Schroeder et al., 2018 appear twice). Check numbering consistency after editing. | We thank the reviewer for their careful reading of the References. This should now be corrected. |

Reviewer #3:

| The cohort for this study is relatively small, with only 19 PSP participants; however, the study is clearly and concisely defined, and as is detailed in the submission, due to the lack of research in this field, it is highly valuable and relevant in terms of person-centered care and a defined need for more specialised interventions for individuals who are disproportionately affected by PTSI’s. … The use of participants' quotes is always a welcome addition and authentically supports the submission. | We appreciate the reviewer’s kind words |
| --- | --- |
| In terms of effectiveness, I question the measure of this, as is stated in line 465 “These different conceptions of effectiveness meant that how the program was seen to support effectiveness varied considerably.” I would encourage further exploration into empowerment, self-efficacy, self-worth & connectedness. | We agree that effectiveness might be better understood in different terms, and we appreciate the suggestions provided. We have attempted to integrate some of this terminology into the Discussion, but were somewhat limited with other revisions to stay within reasonable word length. |
| Sustainability of the study is also unclear. It would be valuable to add what happens after this pilot and how ongoing support for participants could be integrated into future efforts. | This is a fair point. We have added a recommendations section to the paper that focuses on future support. |

Reviewer #4:

| This is a timely and well-executed qualitative evaluation of a pilot mental health program for Public Safety Personnel (PSP). … 4. Balanced Discussion: The discussion section effectively situates the findings within existing literature and thoughtfully acknowledges the study's limitations. | We appreciate the reviewer’s kind words |
| --- | --- |
| 1. Addressing Cumulative Trauma: A participant noted the program felt geared toward singular traumatic events rather than cumulative trauma. The Discussion section could be strengthened by exploring how future programs might better accommodate the cumulative nature of trauma prevalent among PSP. | This is a helpful suggestion and we included a paragraph to this effect. |
| 2. Elaborating on Virtual Delivery Challenges: The paper highlights the trade-off between accessibility and connection in virtual therapy. It would be beneficial to discuss potential solutions, such as hybrid models or specific virtual facilitation techniques, to mitigate the limitations of online group sessions. | This is also helpful, and we have added a section on relationality in virtual therapy. We did not add suggestions for hybrid models, as the choice for virtual only was intentional by the program based on its province-wide accessibility. Although other programs might consider it. |
| 3. Refining Group Composition Recommendations: The finding that group composition (e.g., rank, gender) impacts engagement is critical. Highlighting this as a specific, actionable recommendation in the conclusion would be valuable for program designers. | We added this to our Discussion section. |
| 4. Emphasizing Patient-Centered Discharge: The abruptness of the discharge process was a significant point of distress for participants. The authors' suggestion for a tapered transition is excellent and deserves greater emphasis as a concrete recommendation to prevent an erosion of progress. | We now address this in two ways. We tie this into continuity of care in the Discussion and also make a recommendation for follow-up with PSP post discharge. |

Reviewer #5:

| This is a generally well-written exposition of a pilot MH program to treat public safety personnel (PSP) with post-traumatic stress injury. It is very detailed in its presentation of narrative interviews of PSP and the persons trained to conduct their therapy program. While overall an interesting article, and useful as an early report on specialty treatment programs for first responders, there are areas that are too verbose and other areas lacking in sufficient detail, as follows. | We appreciate the reviewer’s kind words and helpful recommendations |
| --- | --- |
| The concept of post-traumatic stress injury (PTSI), as used in the title and throughout the MS, is never defined. The authors never explicitly state that it is different from syndromal PTSD, and the PCL-5 rating scale the authors used does have a threshold for civilian PTSD. So, how many of the PSP participants had full, syndromal PTSD and how many did not (e.g., subsyndromal PTSD – Other Specified Trauma- and Stressor-Related Disorder in DSM-5)? How did they differ in traumatic event exposure, treatment response, etc.? | As we mention with another reviewer, we initially decided to use PTSI as an inclusive term, but given that the program explicitly is designed and open to PSP with a formal diagnosis of PTSD, we have changed our terminology where appropriate or necessary.  All participants were diagnosed with full, syndromal PTSD.  We have no information unfortunately in how they differed in terms of exposure, response, etc. We did add a newly published quantitative reference on the program’s success with return-to-work. |
| Abstract: The last sentence, lines 48-51 is vague – what were some of the conflicting goals? If the PSP made no progress by established metrics but still were better able to manage their PTSI (a vague statement in itself), then were the metrics off the mark? Better to remove this sentence and leave it for the Discussion, where the details can be presented. | This is a good point and we have removed the sentence. |
| Introduction: Line 59: “disproportionately” – vs whom? Suggest eliminating this sentence and putting ref 5 with previous sentence | We agree that it was not clear, but we felt removing it disrupted the flow. We have amended for clarity. |
| . Line 60: “considerable barriers to treatment” – a provocative statement; give some examples of a “considerable barrier.” | We now explicitly state that examples are provided afterwards. |
| Line 67: PSP were on occupational leave for PTSI – was this syndromal PTSD? See comments in paragraph 2 above. | Yes, see comment above |
| Note that line 95 refers to PTSD, which is mentioned a few more times throughout the MS. | Similarly, see comment above |
| Lines 97-123: These paragraphs are redundant to earlier sections of the Introduction and can be integrated with the earlier sections. | We reviewed this content and while the introduction outlines the incidence, causes and effects of PTSD, this section characterizes the treatment programming needs for individuals with PTSD. Therefore we have retained this text separate from the introduction. Perhaps the other revisions have provided more distinction. |
| Line 126: Move (FRMH) up to line 125 after First Responder Mental Health. | We have changed this |
| Methods: Line 143: Participants (plural). Line 150: Delete initial “the.” Line 166: Suggest adding “residential” after “main.” | Corrections were made |
| Results: Of those invited to participate, what % do the 11 providers and 19 PSP represent? How do they compare demographically to the total invited sample? Is there some selection bias here? And, what were the psychotropic medications the PSP were taking? A table indicating the demographics of both groups, including PSP comorbidities and medications, would be helpful. | This was a helpful suggestion for clarity. We have added a table of demographics. Unfortunately, we only collected limited information from participants, we have no clinical knowledge of participants (e.g., on medication use or comorbidities).  We have added all this to our Limitations section around sampling and bias in our Discussion. |
| Line 219: “…challenges for PSP with complex needs…” is a provocative but vague statement. What kind of challenges and what kind of complex needs? | We have rewritten this to make it clearer |
| Overall, the Results section is wordy. The participant quotes are useful, but there are a lot of them, and many contain redundant concepts. The quotes thus could be condensed. Following are three examples; please review the others and condense as much as possible.  … | We appreciate the need to be concise. Following the reviewer’s suggestion, we have condensed or removed quotations where we felt this was feasible, as well as shortened the Results section. This cut the space by about 15-20%. |
| Lines 367-369: Have you considered having the staff go on calls with the PSPs to have first-hand knowledge of the challenges of being a particular type of PSP? | This could be valuable for this and other treatment programs. We now point this out in our Discussion. |
| Lines 375-379: Please detail the explanations of confidentiality that were given to the PSPs at the beginning of the program. | We have added to the Methods section on who has access to patient data, but as we did not design the program, we do not know exactly what PSP are told by their therapists. |
| Line 464: “…different conceptions of effectiveness…” is a vague statement. What were the different conceptions and the resulting participant perceptions of effectiveness. Give some examples, please. | This is a good point and we have added examples. |
| Overall, the Results section is very long. In addition to condensing the many quotes, please consider condensing the text as well. | See comment above |
| Discussion: Line 531: Delete “Appropriate” and start sentence with “Treatment”. (Appropriate vs what? Inappropriate?) | We have made this change |
| Lines 553-554: How many PSPs returned to work? This might be added to the table and referred to in this part of the Discussion. | We have added a paragraph to the discussion in our reflection on effectiveness focusing on the “success” of the program, referring to the quantitative evaluation that was published elsewhere. We do not have the numbers for the specific PSP who participated in our study, as our return-to-work data were anonymous, and interviews took place shortly after program completion. |
| Lines 599-600: The PCL-5 is a subject-rated questionnaire and can be biased depending on the circumstances (e.g., over-scored by veterans seeking service-related monetary compensation), so that clinician-rated questionnaires; e.g., the CAPS, might serve the program better. Perhaps consider this under Future Research. | We have added to the Methods section that clinicians make separate reports. |

Reviewer #6:

| The manuscript offers a valuable perspective on mental health programs for public safety personnel with post-traumatic stress injury. It is well-detailed, technically sound, and demonstrates potential for replicability. However, the authors should consider addressing the following points to further strengthen the manuscript: | We appreciate the reviewer’s kind words and helpful suggestions |
| --- | --- |
| The authors should explicitly list the specific events being referenced in the statement, “Members of the general public are exposed to only three of those events.” | This was a poorly phrased sentence from us. We have amended to clarify what we meant, that it’s 3 in total on average. |
| Citation Needed (Line 92–94) A supporting citation is required for the claim made in this section to enhance credibility and academic rigor. | We have added a citation |
| Recruitment and Sampling Details (Line 178) More detailed information on the participant recruitment process and sampling methodology is recommended for improved transparency. | We have expanded this section, now explicitly stating that we did self-selection sampling and when sampling ended. |
| Theoretical Framework Visualization (Line 157) If possible, a diagrammatic representation of the theoretical framework would enhance reader comprehension. | We agree and normally we would add such a representation. The framework is not that fancy though, it’s merely an attempt to synthesize other theories into three overarching categories. There’s no description of how categories affect each other and such. |
| Clarification on Data Collection Tools It would improve clarity if the authors explicitly mention that two separate data collection tools were used, describe each tool, indicate whether they were adapted from previous studies, and state whether they were validated. | We have attempted to provide clarity on the design of the two interview guides, one for each participant group. They were not validated. They were iteratively reviewed by the full study team. |
| The authors should ensure that the conclusion remains objective and is explicitly aligned with the stated objectives of the study. The final statements should reflect the study’s findings without overgeneralization or speculative claims. | We agree on the importance of this. We have rephrased our Conclusion which is now hopefully in line with the reviewer’s concern. |
